# A Relação NUS/Cr Confere Pior Prognóstico em Todos os Espectros de Fração de Ejeção?

**DOI:** 10.36660/abc.20230107

**Published:** 2023-03-27

**Affiliations:** 1 Hospital Barra D’Or Rio de Janeiro RJ Brasil Hospital Barra D’Or, Rio de Janeiro, RJ – Brasil; 2 Universidade Federal do Rio de Janeiro Rio de Janeiro RJ Brasil Universidade Federal do Rio de Janeiro, Rio de Janeiro, RJ – Brasil

**Keywords:** Insuficiência Cardíaca, Nitrogênio da Ureia Sanguínea, Volume Sistólico/fisiologia, Doenças Renais/complicações, Hospitalização, Mortalidade

A ativação neuro-hormonal é uma das características fisiopatológicas mais relevantes na insuficiência cardíaca (IC), promovendo efeitos deletérios em longo prazo que contribuem para o desenvolvimento da síndrome cardiorrenal. Essa hiperativação está associada ao prognóstico de insuficiência cardíaca aguda; portanto, é plausível supor que sua atividade esteja associada ao prognóstico de insuficiência cardíaca.^1^

A atual classificação universal da insuficiência cardíaca, atualizada em 2021, modificou a classificação da IC de acordo com a fração de ejeção (FE), substituindo a FE intermediária (ICFEmr) pela FE levemente reduzida para aqueles com FE de 41% a 49%, mantendo a classificação de fração de ejeção reduzida (ICFEr) para aqueles com FE menor que 40% e preservada (ICFEp) para pacientes com FE acima de 50%.^2^ O sistema de classificação mais utilizado atualmente é baseado nessa categorização dos pacientes em grupos com base na fração de ejeção do ventrículo esquerdo (FEVE), e esse modelo tornou-se o principal modelo utilizado pelas diretrizes para fornecer recomendações de tratamento.^3^

IC e disfunção renal frequentemente coexistem, compartilhando muitos fatores de risco (diabetes, hipertensão e hiperlipidemia), contribuindo para piorar o prognóstico.^4,5^ A síndrome cardiorrenal é caracterizada pela piora da função renal durante a internação por insuficiência cardíaca, ou logo após a alta, apesar da melhora sintomática com tratamento com diuréticos e manutenção de volume intravascular adequado.^5^ A creatinina, o nitrogênio ureico no sangue (NUS) e a taxa de filtração glomerular (TFG) são os marcadores tradicionalmente utilizados na prática clínica para avaliar a IC descompensada. A atividade neuro-hormonal na insuficiência cardíaca afeta muito a magnitude da reabsorção do NUS. Portanto, a relação NUS para creatinina (NUS/Cr) é geralmente considerada uma métrica de atividade neuro-hormonal relevante.^6^

Assim, o estudo “Relação entre NUS/Cr e prognóstico da IC em todo o espectro da fração de ejeção”^7^ forneceu informações importantes sobre esse tema. Este estudo retrospectivo incluiu 2.255 pacientes com IC sintomática (NYHA classe III-IV) que mediram NUS e creatinina na admissão. A relação NUS/Cr foi avaliada de forma dicotomizada (ponto de corte = 25,5) em subgrupos de acordo com a fração de ejeção (FE). Os resultados do estudo foram definidos como reinternação por IC, morte cardiovascular e morte por todas as causas, avaliadas em 3, 12 e 24 meses, respectivamente.

Na população de FE reduzida, os pacientes com relação NUS/creatinina elevada (>25,5) exibiram um risco aumentado de morte cardiovascular em 3, 12 e 24 meses e morte por todas as causas em 3 meses. Por outro lado, em pacientes com ICFEmr, não houve diferença significativa nos resultados. Finalmente, em pacientes com ICFEp, uma alta relação NUS/Cr aumentou o risco de reinternação por IC aos 12 e 24 meses e o risco de morte por todas as causas.

Estudos anteriores já demonstraram o valor prognóstico da relação NUS/creatinina em pacientes com IC, principalmente naqueles com disfunção renal.^8,9^ Além disso, Parrinelo demonstrou que essa relação pode estar correlacionada com o grau de congestão sistêmica, justificando seu aumento em pacientes internados com IC descompensada.^10^ Por fim, nesse mesmo cenário, a presença de relação NUS/Cr elevada na admissão identifica pacientes com probabilidade de recuperação da função renal com o tratamento,^8^ demonstrando que diversos mecanismos podem explicar como essa relação impactará na mortalidade.

Alguns estudos não demonstraram diferença na mortalidade de acordo com a classificação baseada na fração de ejeção em curto e longo prazo.^11^ Entretanto, sabemos que existem diferenças fisiopatológicas nesses espectros de FE, o que pode justificar o valor prognóstico da relação NUS/Cr, principalmente na IC com FE reduzida, uma vez que esses pacientes apresentam maior congestão sistêmica. Esse fator justificaria o aumento dessa relação.

O papel da relação NUS/Cr como uma métrica da atividade neuro-hormonal, portanto, precisa ser mais estudado. É necessário entender seu papel no espectro de classificação da fração de ejeção e toda a prática clínica, justificando assim seu uso rotineiro na prática clínica.
