# Supplementary material for: A Relação NUS/Cr Confere Pior Prognóstico em Todos os Espectros de Fração de Ejeção?
Source: Arq Bras Cardiol. 2023 Mar 27;120(3):e20230107. [Article in Portuguese] doi: 10.36660/abc.20230107 (PMC10263454; doi:10.36660/abc.20230107)
Supplement: Translation

# Translation: Does the BUN/Cr Ratio Confer a Worse Prognosis in All Ejection Fraction Spectra?

**Affiliations:** 1 Rio de Janeiro RJ Brazil Hospital Barra D’Or, Rio de Janeiro, RJ – Brazil; 2 Rio de Janeiro RJ Brazil Universidade Federal do Rio de Janeiro, Rio de Janeiro, RJ – Brazil

**Keywords:** Heart Failure, Blood Ureia Nitrogen, Stroke Volume/pysiology, Kidney Diseases/complications, Hospitalization, Mortality

Neurohormonal activation is one of the most relevant pathophysiological features in Heart Failure (HF), promoting long-term deleterious effects that contribute to the development of cardiorenal syndrome. This hyperactivation is associated with the prognosis of acute heart failure; therefore, it is plausible to hypothesize that its activity is associated with the prognosis of heart failure.^1^

The current universal classification of heart failure, updated in 2021, modified the classification of HF according to ejection fraction (EF), replacing the mid-range EF (HFmrEF) with mildly reduced EF for those with an EF of 41% to 49%, maintaining the classification of reduced ejection fraction (HFrEF) for those with EF less than 40% and preserved (HFpEF) for patients with EF above 50%.^2^ The most used classification system today is based on this categorization of patients into groups based on left ventricular ejection fraction (LVEF), and this model has become the main model used by guidelines to provide recommendations for treatment.^3^

HF and renal dysfunction frequently coexist, sharing many risk factors (diabetes, hypertension, and hyperlipidemia), contributing to worsening the prognosis.^4,5^ The cardiorenal syndrome is characterized by worsening renal function during hospitalization for heart failure, or soon after discharge, despite symptomatic improvement by treatment with diuretics and maintenance of adequate intravascular volume.^5^ Creatinine, blood urea nitrogen (BUN), and glomerular filtration rate (GFR) are the markers traditionally used in clinical practice to assess decompensated HF. Neurohormonal activity in heart failure greatly affects the magnitude of BUN reabsorption. Therefore, the BUN to creatinine ratio (BUN/Cr) is generally regarded as a relevant neurohormonal activity metric.^6^

Thus, the study “Relationship between BUN/Cr and prognosis of HF across the full spectrum of ejection fraction”^7^ provided important information on this topic. This retrospective study included 2255 patients with symptomatic HF (NYHA class III-IV) who measured BUN and creatinine on admission. The BUN / Cr ratio was evaluated dichotomized (cutoff point = 25.5) in subgroups according to the ejection fraction (EF). Study outcomes were defined as HF readmission, cardiovascular death, and all-cause death, assessed at 3, 12 and 24 months, respectively.

In the reduced EF population, patients with an elevated BUN/creatinine ratio (>25.5) exhibited an increased risk of cardiovascular death at 3, 12, and 24 months and all-cause death at 3 months. On the other hand, in patients with HFmrEF, no significant difference was observed in outcomes. Finally, in patients with HFpEF, a high BUN/Cr ratio increased the risk of re-hospitalization for HF at 12 and 24 months and the risk of death from all causes.

Previous studies have already demonstrated the prognostic value of the BUN/creatinine ratio in patients with HF, especially those with renal dysfunction.^8,9^ In addition, Parrinelo demonstrated that this relationship might be correlated with the degree of systemic congestion, justifying its increase in patients hospitalized with decompensated HF.^10^ Finally, in this same scenario, the presence of a high BUN/Cr ratio on admission identifies patients likely to show recovery of renal function with treatment,^8^ demonstrating that several mechanisms can explain how this ratio will impact mortality.

Some studies have shown no difference in mortality according to the classification based on the ejection fraction in the short and long term.^11^ However, we know that pathophysiological differences exist in these EF spectra, which may justify the prognostic value of the BUN/Cr ratio, especially in HF with reduced EF, since these patients exhibit greater systemic congestion. This factor would justify the increase in this ratio.

The role of the BUN/Cr ratio as a metric of neurohormonal activity, therefore, needs further study. It is necessary to understand its role in the spectrum of ejection fraction classification and all clinical practice, thus justifying its routine use in clinical practice.
